# A Shocking Affair.—Death in a Dentist’s Office

**Published:** 1870-04

**Authors:** 


We take the following from the *Berkshire Courier:*

A Shocking Affair.—Death in a Dentist's Office.—On Saturday afternoon this community was shocked to hear that a lady had died in the office of Dr. W. W. Rice, from the effects of chloroform administered previous to having some teeth extracted. The lady—Mrs. Bradford C. Foote of Sheffield—having suffered for nearly a week with aching teeth, came up with her husband to have them out, and insisted upon taking chloroform. Her husband and Dr. Rice both tried to dissuade her from taking it, the former telling her that he was afraid she might die from its effects. She replied that she "could not die younger," but that she could not endure the pain of having the teeth pulled without it. Dr. Rice then proposed to her to have the one out which had become ulcerated, and leave the eleven others she proposed to have pulled, until another time. But she would not agree to it, and so about 2 P. M., Dr. Parks was called in and the chloroform administered. We give his report of the sad affair:

The sudden death of Mrs. Foote on Saturday afternoon, having awakened the sympathy of the entire community, and given rise to many inquiries and rumors concerning it, I send you the following report of the case which you are at liberty to publish as a matter of public interest. Mrs. Foote of Sheffield, aged 36 years, called on Saturday, February 12, at the dental office of Dr. Rice in this village for the purpose of having some teeth extracted; I was requested by the husband to administer chloroform. He stated that she had once before taken it without ill effect. Mrs. F. being apparently a woman in good health, I consented to administer it. It was given in the usual manner, by pouring a small quantity upon a napkin, held at a little distance from the face in order not to interrupt a sufficient supply of atmospheric air. No unusual effect was produced until after twelve teeth were extracted when, for the moment, she manifested signs of returning consciousness, and while leaning forward in the act of retching or vomiting, after two or three ineffectual attempts she suddenly expired. I was supporting the head at the time with my fingers pressing upon the temporal artery, the pulsation of which I could distinctly feel. The first intimation of danger perceived was the sudden cessation of the pulsation, when, after a few gasps, she ceased to breathe. I immediately placed her in a horizontal position, and kept up an artificial respiration for half or three quarters of an hour, when, perceiving the hopelessness of the attempt, further efforts were abandoned. It is to be regretted that her friends were not willing that a post mortem examination should be had, as there is some reason for suspecting the existence of an organic disease of the heart. Be this as it may, however, there is no doubt that death resulted from asthenia of the heart induced by the inhalation of chloroform, and the existence of an organic disease of this organ would only have contributed to the danger of such a result. W. H. Parks, M. D.

It is truly to be regretted that a post mortem examination was not had to definitely settle the cause of her death. There was an opinion current that the blood, from the lacerated gums, running down her throat had caused her to strangle, and being under the effects of the anæsthetic, she could not raise it, as it appears she tried or attempted to do. We should suppose a stomach-pump would have removed that difficulty. As all efforts at resuscitation had been abandoned, the body was put into a coffin and taken to Sheffield at five o'clock. There were wild rumors afloat that upon opening the coffin, after reaching home, the body was turned, and also that one hand was found up to her head; but Mr. Foote informs us that these rumors were without foundation. The head was slightly turned, but no more than could be expected from the bad roads and snow drifts the team had to go over. The body, however, was quite warm—almost as much so as in life—and remained so for some hours.

A post mortem examination of the body was had, but failed to reveal any cause of her death. The organs were found unaffected by disease, save a small, but unimportant spot upon the liver, and the physicians report that there was nothing apparent in the examination to have occasioned the fatal result. This at least proves the danger of taking chloroform, even by healthy people, and the warning will undoubtedly be heeded for some time to come.

